# Ovarian small cell carcinoma by ovarian torsion feature: A cytopathology challenging case

**DOI:** 10.1016/j.ijscr.2022.107337

**Published:** 2022-06-22

**Authors:** Fatemeh Keikha, Alireza Hadizadeh, Setareh Akhavan, Fatemeh Nili, Arefeh Eshghinejad, Marjan Ghaemi

**Affiliations:** a Vali-E-Asr Reproductive Health Research Center, Family Health Research Institute, Tehran University of Medical Sciences, Tehran, Iran; b School of Medicine, Tehran University of Medical Sciences, Tehran, Iran

**Keywords:** Ovarian cancer, Small cell carcinoma, Ovarian torsion

## Abstract

**Introduction and importance:**

Ovarian small cell carcinomas are a rare type of ovarian cancer that is highly aggressive and consists of two distinct types the hypercalcemic type (SCCOHT) and pulmonary type (SCCOPT).

**Case presentation:**

A 23 years old girl was admitted to the emergency room with the presentation of acute abdomen. The ultrasound and Magnetic resonance imaging revealed a right adnexal huge mass with adnexal torsion. In laparotomy, she underwent unilateral salpingo-oophorectomy due to ovarian torsion and possible malignancy. The histopathological evaluation was challenging and was finalized by a team of pathologists as hypercalcemic small cell carcinoma. She refused reoperation and unfortunately relapsed during chemotherapy and died 6 months after the initial diagnosis.

**Clinical discussion:**

Conclusion: We do not yet have comprehensive information on small cell ovarian cancer. Cytopathology diagnosis is still challenging and the treatments are not usually effective. Further clinical trials and studies are recommended to find appropriate treatments for these patients.

## Highlights

• Ovarian small cell carcinomas are a rare type of ovarian cancer with high mortality.
• Cytopathology diagnosis is still challenging and the treatments are not usually effective.
• Further clinical trials and studies are recommended to find appropriate treatments in these patients.

## 1 Introduction

Ovarian cancers are one of the leading causes of mortality among women around the world and are the third most prevalent gynecological cancers following cervical and uterine cancers. Ovarian small cell carcinomas (SCCs) are a rare type of ovarian cancer that is highly aggressive [1]. SCCs arise from neuroendocrine organs; therefore, they could involve and occur in various sites including the lungs, gastrointestinal system, and in a few instances, they have occurred in ovaries either primarily or secondarily to another site [2], [3].

They affect nearly 1 % of ovarian cancer and consist of two types including small cell carcinoma of the ovary, hypercalcemic type (SCCOHT), and small cell carcinoma of the ovary pulmonary type (SCCOPT). The clinical manifestations usually include palpable mass and abnormal uterine bleeding; however, like other types of small cell carcinomas paraneoplastic features such as hypercalcemia may be present [1], [4]. Currently, there is no consensus on the clinical approach and treatment of small cell carcinomas and a combination of surgical treatment and chemotherapy with radiotherapy is used for these patients [4], [5].

## 2 Case presentation

A 22-year-old, single girl presented at the emergency room with localized hypogastric colicky pain that was started a week prior. She studied in the university. Her menstrual cycles were regular and her last menstrual period was 2 weeks before admittance. She stated that she experienced intermittent episodes of low-level fever for the past 3 days. She did not use any drugs. No history of familial genetic diseases was reported by her and her parents. On physical examination, the patient was mildly tachycardic (PR: 94) but was not febrile or hemodynamically unstable. Her abdominal examination revealed tenderness in the lower left quadrant and hypogastric area. Laboratory tests including complete blood count (CBC), beta HCG, urine analysis, and blood sugar (BS) as well as abdominal ultrasonography were requested with suspicion of ovarian torsion.

**Fig. 1:**
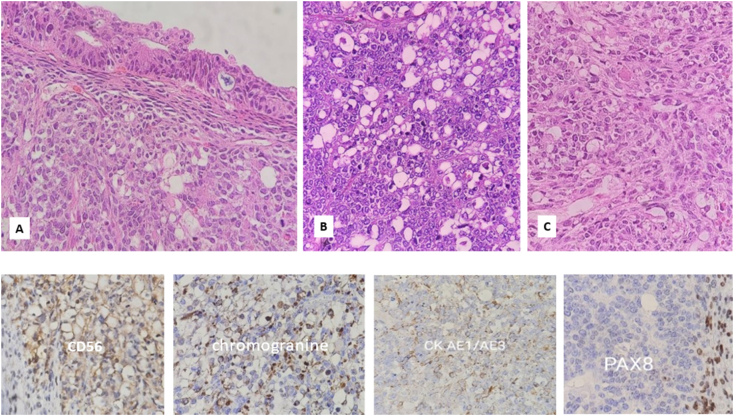
(A-C): MRI scans showcasing (71 × 99 mm) necrotic mass in left adnexa (orange arrows). (For interpretation of the references to color in this figure legend, the reader is referred to the web version of this article.)

The sonographic evaluation revealed a normal uterus and right ovary and a 90 × 96 mm mass with heterogeneous echogenicity was observed with a high suspicion of left adnexa and ovarian torsion. Magnetic resonance imaging (MRI) was also requested. This further assessment revealed a 71 × 99 mm necrotic mass which made malignancies and ovarian torsion the more probable causes (Fig. 1).

After consulting with an oncologist gynecologist, she underwent a laparotomy by a gynecology oncologist. During the surgery with a midline incision, a necrotic 12 × 94 mm mass in the posterior Cul de Sac with adhesion to the intestines was seen. She underwent a left salpingo-oophorectomy. The adhesion bonds from the intestine to the ovaries were resected and the samples from abdominal fluid and omentum were obtained for further analysis. In the abdominal explore, no significant abnormalities were detected except the tip of the appendix which was also inflamed and an appendectomy was performed either.

**Fig. 2:**
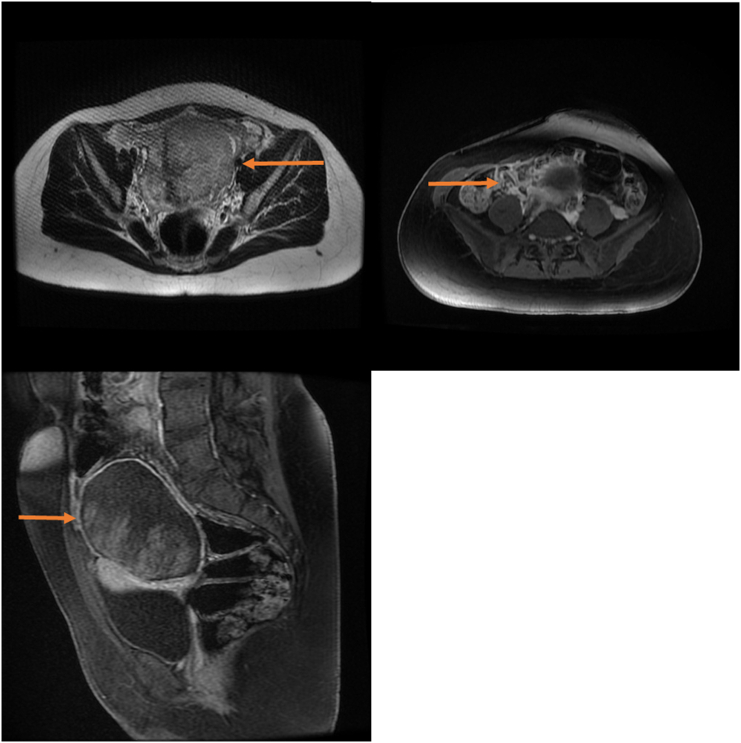
(A-C) Microscopic examination shows sheet of large atypical cells with round to oval pleomorphic vesicular nuclei. Occasional rhabdoid cells, high mitotic activity and areas of necrosis are also seen. In some foci, stratified mucinous epithelial lining are evident. IHC study reveals focal positive reaction in with CK AE1/AE3, CD56 and chromogranin (D-G).

The histopathological evaluation of the morphology of the sample was challenging and was finalized by a team of pathologists working in the field of gynecological oncology. They stated hypercalcemic small cell carcinoma as a diagnosis. The images of the pathology slides are listed in Fig. 2.

Serum tumor marker result is listed in Table 1. No malignant cells were found in the cytology of abdominal fluid. Although the sample from the appendix turned out to be free from the tumor the one from the omentum was involved. The immunohistochemistry (IHC) result is listed in Table 2.

**Table 1:** 

Test	Result	Normal range
CEA (carcinoembryonic antigen)	4.91 ng/mL	<5
CA-125	504.6 U/mL	<35
CA 15-3	25.2 U/mL	<31.3
CA 19-9	28.22 U/mL	<37
AFP (alpha-fetoprotein)	0.46 ng/mL	<6.0 in non-pregnant
Beta-HCG	0.43 mIU/mL	Negative <10
HE4 (human epididymis protein)	41.1 Pmol/L	<70
ROMA (risk of ovarian malignancy algorithm)	5.9 %	Low risk <11.4

**Table 2:** Immunohistochemistry result of the mass.

Tumor marker	Results
SALL4 (Sal-like protein 4)	Negative
AFP (alpha-fetoprotein)	Negative
Glypican-3	Weekly positive in some tumor cells
CD30	Negative
CK7 (cytokeratin)	Negative
PAX8	Positive in spindle cell components and glandular cells
S100	Negative
Chromogranin	Positive
Synaptophysin	Negative
GFAP (glial fibrillary acidic protein)	Positive in some tumor cells
Inhibin	Negative
Calretinin	Negative
Cytokeratin AE1/AE3	Negative

The case was consulted with the tumor board of the university and also with gynecooncologists from USA and UK. While some believed that the patient should undergo another surgery, other consultations suggested chemotherapy since small cell carcinoma of the ovary is associated with a poor prognosis and the medical treatment should not be delayed. The patient also stated that she would choose chemotherapy over surgery. The disease relapsed during chemotherapy with severe side effects that made her stop treatment and be observed and received palliative therapy. Unfortunately, the general condition and symptoms made worse and she died 6 months after the initial diagnosis.

This report was written based on the SCARE 2020 Guideline for reporting the surgical case report [6]. This study has approval by institutional review board affiliated to Tehran University of Medical Sciences.

## 3 Discussion

Small cell carcinomas are a rare gynecological malignancy that constitutes 1 % of all ovarian carcinomas [4]. Depending on the histopathological findings and the clinical presentations and features, the treatment plans could substantially differ [2], [7], [8]. While the hypercalcemic type affects the younger women and is more resistant to chemo and radiotherapy, the pulmonary type affects older women and is susceptible to chemotherapy. On the other hand, the hypercalcemic type is more associated with paraneoplastic symptoms [4].

Both these categories of small cell carcinomas are immune positive like other types of small cell cancers that involve other sites [4]. The most prominent feature of paraneoplastic manifestations is hypercalcemia which becomes symptomatic in 10 % of the cases [9]. This is as a result of PTH-related protein secretion by the cancer cells. Another notable feature of small cell carcinoma is that the tumor markers, which are particularly helpful in diagnosing other epithelial ovarian cancers, do not help diagnose these cancers especially if they occur during pregnancy [4], [7], [8], [9].

In regards to treatment, while surgery can remove the mass within the ovaries, we assume that a second look at abdominal organs, fluids, and peritoneum could markedly improve survival rates as the surgical evaluation can detect gross involvements in all sites. It has been suggested that as the chemo sensitive phase of both types of small cell carcinomas is transient, an intensive multi-drug protocol for chemotherapy should be taken. The recommended regimens include VPCBAE therapy consisting of vinblastine (V), cisplatin (P), cyclophosphamide (C), bleomycin (B), doxorubicin (A), and etoposide (E) administered on a 3-week schedule or (PAVEP) consisting of cisplatin (P), doxorubicin (A), etoposide (V), and cyclophosphamide (EP). In extensively affected cases the use of radiotherapy has also been suggested [2], [10].

SCCOHT is currently classified in the miscellaneous group of ovarian cancers in 2020 WHO classification of female genital tract tumors. Due to uncertain histogenesis and overlapping morphology and IHC findings with other types of ovarian tumor, the diagnosis is usually difficult [11], [12].

In our case, germ cell tumors including yolk sac and embryonal carcinoma were the most possible differentials which were excluded by the negative IHC results for SALL4, CD30, Glypican 3 and AFP (alpha-fetoprotein). Epithelial carcinomas were also excluded based on the negative results for EMA (Epithelial membrane antigen) and PAX8. Considering negative results for inhibin and calretinin, juvenile granulosa cell tumors and other types of sex cord stromal tumors were also excluded. Metastatic malignant melanoma, lymphoma and PNET (pancreatic neuroendocrine tumor) were also ruled out based on negative results for S100, LCA (Leukocyte common antigen) and CD99, respectively.

There were some similar previous reports. Gupta et al. reported a middle-age woman with abdominal pain that was diagnosed by an interdisciplinary team including a gynecologist, radiologist and pathologist. The tumor was SCCOPT with a poor outcome [13]. Similarly, Pressey et al. presented a young woman with abdominal pain and bloating with the diagnosis of SCCOHT. They suggested limited surgery (salpingo-oophorectomy) and chemoradiotherapy. The treatment was followed by stem cell transplant as a novel treatment modality [14].

Diffuse expression of WT1 (Wilms' Tumor) is a useful diagnostic test in these cases, which was negative in our case. Based on overall histopathological findings and IHC results, by excluding other more common differential diagnoses, SCCOHT was suggested. Over 95 % of the SCCOHT, exhibit mutations of SMARCA4 (SWI/SNF Related, Matrix Associated, Actin Dependent Regulator of Chromatin, Subfamily A, Member 4) gene and loss of this protein in IHC study. But these confirmatory tests were not available in our center [11], [12].

The survival is not good with only 33 % in the early stages. Although the patients advanced disease rarely survived. It seems the new treatment is promising. The patient's hematologic transplant procedure following chemotherapy had better survival compared to those cases who that received just chemotherapy [15].

In regards to the presented case, while our first suspicion was ovarian torsion, we also suspected that malignancy could cause such extensive destruction of ovaries. Meanwhile, during the surgery, we assessed all abdominal organs and took samples as not only does this approach provide a better grading, but also, it does help remove any gross metastasis.

## 4 Conclusion

We do not yet have comprehensive information on small cell ovarian cancers. Cytopathology diagnosis is still challenging and the treatments are not usually effective. Further clinical trials and studies are recommended to find appropriate treatments for these patients.

## Declaration of competing interest

The authors have not any conflict of interest.
